# Improved Sensitivity in Large Field of View Multispectral Laser‐Scanning Photoacoustic Microscopy for Measuring Oxygen Saturation In Vivo

**DOI:** 10.1002/jbio.202500378

**Published:** 2025-11-10

**Authors:** Mohsin Zafar, Amir Khansari, Rayyan Manwar, Kamran Avanaki

**Affiliations:** ^1^ The Richard and Loan Hill Department of Biomedical Engineering University of Illinois at Chicago Chicago Illinois USA; ^2^ Department of Dermatology and Pediatrics University of Illinois at Chicago Chicago Illinois USA

**Keywords:** in vivo, microscopy, oxygen saturation, photoacoustic, spectroscopy

## Abstract

Multispectral photoacoustic microscopy (PAM) using stimulated Raman scattering (SRS) has been employed to measure oxygen saturation (sO_2_) in biological tissue. However, laser‐scanning photoacoustic microscopy (LS‐PAM) inherently suffers from low detection sensitivity due to the use of a flat transducer and non‐coaxial alignment of the transducer with the optical scan. Although wide‐field‐of‐view LS‐PAM has been implemented, it typically results in coarser lateral resolution and hence lower sensitivity than existing LS‐PAM systems. Here, we present a wide‐field multispectral LS‐PAM system for measuring sO_2_ in biological tissue. Instead of relying on two discrete wavelengths, our method employs two wavelength groups—a isosbestic group (532 nm and 545 nm) and a deoxyhemoglobin‐dominant group (545 nm and 558 nm). We demonstrate that using these groups improves the signal‐to‐noise ratio (SNR) of the detected signals, leading to more accurate sO_2_ measurements. The performance of this system is validated through both phantom and in vivo studies.

## Graphical abstract

Validation of large field of view multispectral laser‐scanning photoacoustic microscopy (LS‐MS‐PAM) for sO_2_ measurements.

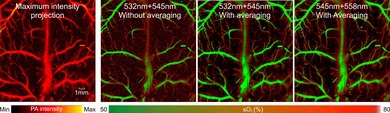

## 1 Introduction

Photoacoustic microscopy (PAM) can be configured as either acoustic resolution‐PAM or optical resolution‐PAM, based on how the focus is achieved. Multispectral PAM systems of either configuration employ multiple wavelengths to selectively target absorbers such as oxyhemoglobin, deoxyhemoglobin, lipids, melanin, and water, offering a comprehensive view of tissue composition [1, 2, 3, 4, 5, 6, 7, 8]. Multispectral imaging is achieved either by using multiple lasers operating at different wavelengths [9] or by harnessing stimulated Raman scattering (SRS) in polarization‐maintaining single mode fibers (PM‐SMF) [8] to generate varied wavelengths from a single laser source [1, 10, 11, 12, 13]. In the latter method, typically, through the SRS effect, researchers have either paired 532 nm with 558 nm [14, 15, 16], or 545 nm with 558 nm [17, 18] to perform measurements of oxygen saturation (sO_2_), where 532 nm and 545 nm are considered as isosbestic wavelengths and 558 nm is considered as the deoxyhemoglobin dominant wavelength.

In one configuration of PAM, an unfocused ultrasound transducer is placed at an angle and held in a fixed position, while focused laser light is scanned point‐by‐point across the field of view (FOV) using a 2D galvanometer. This configuration is often referred to as laser scanning PAM (LS‐PAM) [19, 20, 21]. Because of the use of optical (non‐mechanical) scanning, this technique allows for faster imaging across larger regions, making it advantageous for applications requiring quick, wide‐field scanning [6]. However, LS‐PAM experiences lower signal‐to‐noise ratio (SNR) compared to other PAM setups due to reduced detection sensitivity. This reduced sensitivity arises from several factors: (i) the non‐coaxial arrangement of the optical and acoustic elements, (ii) the use of an unfocused transducer for signal detection, and (iii) the increased distance between the transducer and the imaging area [19, 22]. SNR can in principle be increased by raising the laser energy or performing signal averaging; however, averaging reduces temporal resolution, and increasing laser energy is limited by ANSI safety standards and the damage threshold of optical fibers.

In this study, we adopt a novel method to increase the overall energy of the laser, thereby increasing detection sensitivity. We developed a multispectral LS‐PAM system for sO_2_ measurement and use wavelength groups instead of individual wavelengths: the isosbestic group (532 nm and 545 nm) and the deoxyhemoglobin‐dominant group (545 nm and 558 nm). By combining PA signals from multiple wavelengths, we effectively increase the total input laser energy without exceeding safety or hardware limits, thereby improving detection sensitivity and enhancing SNR. To validate these improvements, we compared SNR from individual wavelengths versus wavelength groups on leaf phantoms and then conducted in vivo mouse brain imaging to assess the accuracy of sO_2_ measurements relative to a traditional wide‐FOV multispectral LS‐PAM approach.

## 2 Materials and Methods

### 2.1 Experimental Setup

**FIGURE 1:**
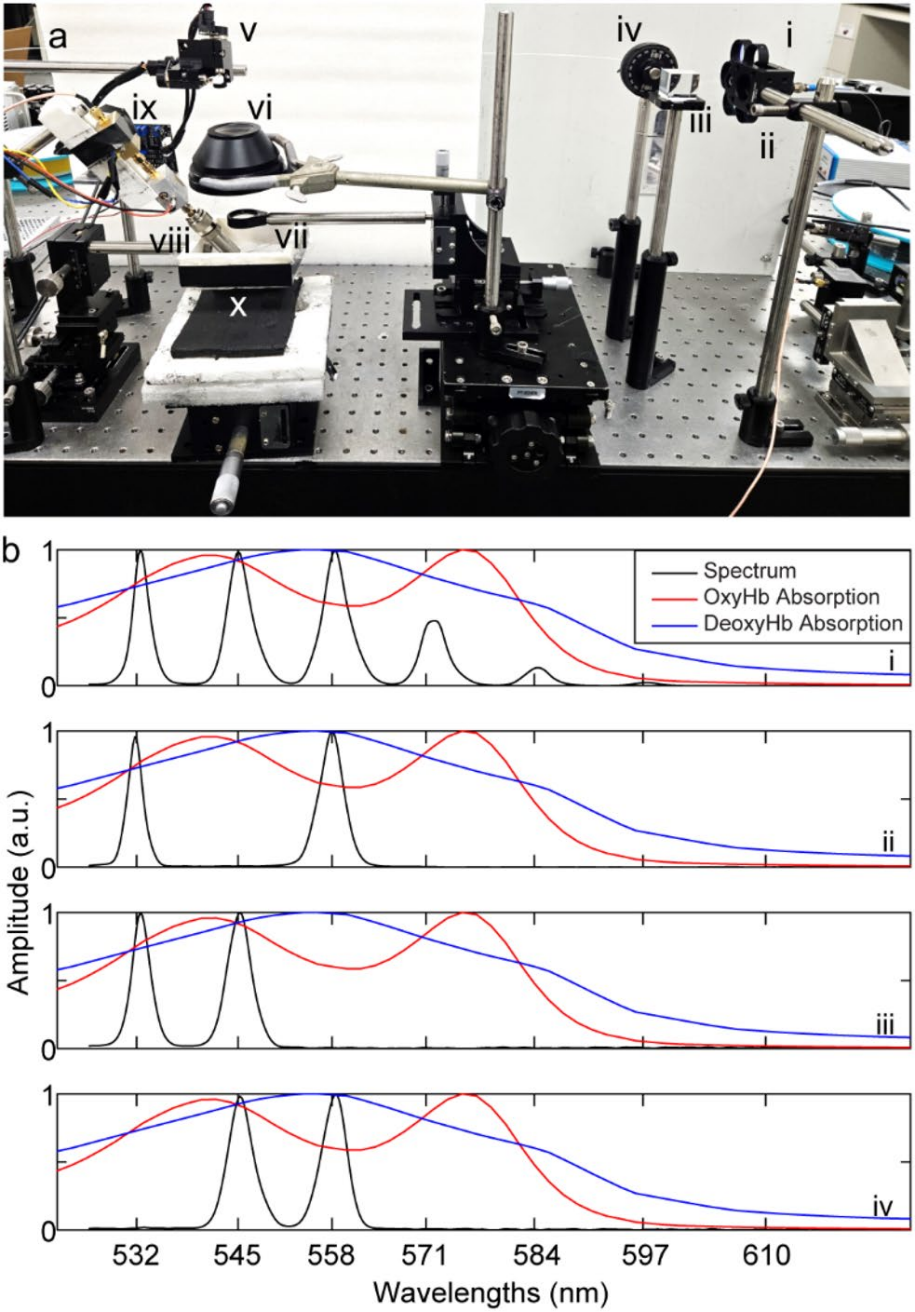
Multispectral LS‐PAM system experimental setup. (a) Photograph of our multispectral LS‐PAM system. The setup includes: (i) Polarization maintaining single mode fiber (PM‐SMF), (ii) filter wheel, (iii) 90:10 beam splitter, (iv) 2 × 1 optical fiber path (v) galvanometer, (vi) F‐theta lens, (vii) doublet lens, (viii) transducer, (ix) amplifiers, (x) water tank. (b) Spectrum Generated: (i) Without filters, (ii) with traditional sO_2_ measurement wavelengths (532 nm and 558 nm) (iii) With optical filter isolating isosbestic group (532 nm + 545 nm) and (iv) With optical filter isolating deoxyhemoglobin dominant group (545 nm + 558 nm).Wavelengths in (iii) and (iv) are used together to perform sO_2_ measurements, marked by green dashed box.

The system configuration of the developed LS‐PAM system is shown in Figure 1a. A pulsed Nd:YAG laser with a central wavelength of 532 nm was used as the optical excitation source with a pulse width of ~13 ns and a pulse repetition rate of 5 kHz. To generate different wavelengths from the 532 nm input, the SRS effect is employed in PM‐SMF similar to the system described in [23]. Briefly, using a combination of half‐wave plates (WPH10E‐532, Thorlabs, Newton, USA) and polarized beam splitter (PBS251, Thorlabs, Newton, USA), the input 532 nm laser light was divided into two polarized components. The p‐polarized component was coupled with a 30‐m‐long PM‐SMF (HB450‐SC, Fibercore, United Kingdom). Through inelastic nonlinear interactions between input photons and the silica fiber, SRS peaks are generated at different wavelengths, increasing in 13 nm increments. A filter wheel (CFW6, Thorlabs, Newton, USA) is used to select these wavelengths at the output of the PM‐SMF. We adjusted the laser energy (see Figure 1b[i]) such that the pulse energy at 532 nm, 545 nm, and 558 nm was closely matched. The output energy for each wavelength was measured using a fast photodetector (PDA36A2, Thorlabs, Newton, USA) to allow pulse‐to‐pulse energy fluctuation compensation. To isolate 532 nm and 558 nm individually, optical filters centered at 532 nm (FLH532‐10, Thorlabs, Newton, USA) and 560 nm (FBH560, Thorlabs, Newton, USA), each with a 10 nm bandwidth, were used (see Figure 1b[ii]). These wavelengths are conventionally employed for two‐wavelength sO_2_ estimation. For grouped‐wavelength‐based sO_2_ measurement, an isosbestic group (532 nm and 545 nm) was selected using a 550 nm short‐pass filter (FESH0550, Thorlabs, Newton, USA) (see Figure 1b[iii]), while a deoxyhemoglobin‐sensitive group (545 nm and 558 nm) was selected using a 550 nm bandpass filter with a ± 12.5 nm bandwidth (86–655, Edmund Optics, New Jersey, USA) (see Figure 1b[iv]). After spectral filtering of multispectral laser light through the filter wheel, the PM‐SMF output path was split through two paths via a 90:10 beam splitter (BS025, Thorlabs, Newton, USA). The 90% path goes straight onto the 2‐dimensional galvanometer (GVS 202‐2D, Thorlabs, Newton, USA) for imaging whereas the 10% path is coupled into a 1 × 2 bifurcated optical fiber (TM200R5F1A, Thorlabs, Newton, USA). Each arm of this fiber is routed through a narrowband optical filter to isolate a specific wavelength within each group. These filtered outputs are then connected to fast photodetectors (PDA36A2, Thorlabs, Newton, USA) to monitor real‐time fluctuations in the optical intensity of each wavelength independently. This configuration allowed dynamic compensation for energy imbalance within each group, ensuring the effective fluence from both wavelengths remained stable and spectrally consistent during scanning.

Triangular signals are generated in LabVIEW and transmitted to the galvanometer via an analog output card (BNC 2110, National Instruments) to enable bidirectional scanning. A frame trigger signal, also simulated in LabVIEW, synchronizes data acquisition with the laser scanning process. The initiation of laser irradiation, raster scanning, and frame triggering was facilitated by a common trigger signal generated by a function generator (ATF20B, USA). The light from the galvanometer is directed onto the imaging object through a sequence of F‐theta lenses (532 nm wavelength, Cloudray, USA) and a doublet lens (AC254‐200‐A, Thorlabs, Newton, USA). Cascading an F‐theta lens with a doublet lens in our multispectral photoacoustic imaging setup enhances field uniformity and minimizes chromatic aberration, improving lateral resolution and wavelength alignment across the scanning range. A 4‐in. by 4‐in. water tank, with its base covered by Saran wrap for optical and acoustic transparency, serves as the imaging environment. The imaging target is positioned against the Saran wrap from below, with ultrasound gel applied in between for acoustic coupling. A total of 40 000 points are scanned within a single frame, with a field of view of 1.2 × 1.2 cm^2^. The detected PA signals are amplified using a low‐noise ZFL‐500LN amplifier and digitized at a sampling rate of 200 MS/s by a 4‐channel data acquisition system (DAQ) (CSE1642, GAGE, USA). The DAQ system was synchronized with the frame trigger signal. More details on system specification and characterization are given in [19, 24, 25, 26].

For sO_2_ measurement, the formula cited in Equation (1).

(1) $${\mathrm{sO}}_{2}=\frac{\epsilon_{\mathrm{de}}\left(\lambda_{\mathrm{de}}\right)\mathrm{PA}\left(\lambda_{i}\right)-\epsilon_{\mathrm{de}}\left(\lambda_{i}\right)\mathrm{PA}\left(\lambda_{\mathrm{de}}\right)}{\left[\epsilon_{i}\left(\lambda_{i}\right)-\epsilon_{\mathrm{de}}\left(\lambda_{i}\right)\right]\mathrm{PA}\left(\lambda_{\mathrm{de}}\right)-\left[\epsilon_{i}\left(\lambda_{\mathrm{de}}\right)-\epsilon_{\mathrm{de}}\left(\lambda_{\mathrm{de}}\right)\right]\mathrm{PA}\left(\lambda_{i}\right)}$$

where $\epsilon_{i}$ and $\epsilon_{\mathrm{de}}$ are the molar extinction coefficients of isosbestic and deoxy‐hemoglobin at different wavelengths (shown in Figure 1), and PA is the PA signal amplitude taken at different wavelengths. $\lambda_{\mathrm{de}}$ is either 558 nm or the deoxyHb group of wavelengths (545 + 558 nm) and $\lambda_{i}$ is either 532 nm or the isosbestic group wavelengths (532 + 545 nm).

### 2.2 Preparation of Animal Model

4‐week‐old BALB mice were used for in vivo imaging of the brain. Prior to imaging, the designated areas of the animals were thoroughly cleansed with sterile saline solution. To ensure optimal acoustic coupling for imaging, a layer of ultrasound gel is applied to the cleaned area. The mice are positioned securely in our imaging system, and a 12 mm × 12 mm region was selected for imaging. Anesthesia was induced using 2% isoflurane via inhalation, followed by maintenance doses ranging from 2% to 3% to keep the mice anesthetized during the procedure. The depth of anesthesia was assessed using a toe pinch reflex test to ensure the animals were adequately sedated. For brain imaging, the scalp is surgically removed in a parasagittal orientation, exposing the brain tissue. Ultrasound gel is applied directly onto the exposed brain region to ensure effective acoustic coupling. After all imaging procedures are completed, the mice are euthanized according to the protocol. All experimental protocols adhered to the guidelines outlined in the Guide for the Care and Use of Laboratory Animals and received approval from the University of Illinois, Chicago Animal Care and Use Committee, ensuring ethical and humane treatment of the animals throughout the study.

### 2.3 Quality Metrics

To evaluate our results, we used four quality metrics: mean signal‐to‐background ratio (SBR), normalized mean square error (NMSE) the structural similarity index measure (SSIM) and normalized cross‐correlation (NCC). SBR (Equation 1) quantifies how much stronger the signal was compared to the background. NMSE (Equation 2) measures the average error between normalized corresponding pixels in the reference image and the test image, effectively capturing the overall deviation in intensity values. SSIM (Equation 3) was used to evaluate the perceptual similarity between two images (a reference image generated using traditional multispectral LS‐PAM versus the one generated by our approach) by assessing two key components: contrast and structural information, Finally, NCC (Equation 4) measures the similarity between two images by evaluating the correlation of their pixel intensities and how well the intensity patterns in one image align with the corresponding patterns in the other. Although SSIM and NCC are both similarity metrics, they focus on different aspects of the image (i.e., SSIM emphasizes perceptual quality by comparing structure and contrast, while NCC focuses on the intensity correlation between images).

(2) $$\mathrm{SBR}=10\log\left(\frac{\;\mu_{\text{Signal}}}{\;\mu_{\text{Background}}}\right)$$

(3) $$\text{SSIM}\left(x,y\right)=\frac{\left(2\mu_{x}\mu_{y}+C_{1}\right)\left(2\delta_{\mathrm{xy}}+C_{2}\right)}{\left(\mu_{x}^{2}+\mu_{y}^{2}+C_{1}\right)\left(\delta_{x}^{2}+\delta_{y}^{2}+C_{2}\right)},$$

(4) $$\text{NMSE}\left(x,y\right)=\sqrt{\frac{\sum_{i=1}^{N}{\left(x_{i}-y_{i}\right)}^{2}\;}{\sum_{i=1}^{N}x_{i}^{2}}}$$

(5) $$\mathrm{NCC}\left(x,y\right)=\frac{\sum_{i,j}\left(x_{i,j}-\mu_{x}\right)\left(y_{i,j}-\mu_{y}\right)}{\sqrt{\sum_{i,j}{\left(x_{i,j}-\mu_{x}\right)}^{2}}\sqrt{\sum_{i,j}{\left(y_{i,j}-\mu_{y}\right)}^{2}}}$$

where *μ*_Signal_ is the mean value of 20 signals in the region of 1 × 1 mm area and *μ*_Background_ is the mean value of 20 signals in the background within the same region. $\mu_{x}$ and $\mu_{y}$ are the mean intensities of images x and y, respectively, $\delta_{x}^{2}$ and $\delta_{y}^{2}$ are the variances of images x and y, respectively, $\delta_{\mathrm{xy}}$ is the covariance between x and y, $C_{1}$ and $C_{2}$ are small constants to stabilize the division, $x_{i,j}$ and $y_{i,j}$ are the pixel values at position $\left(i,j\right)$ in images x and y, respectively and N is the total number of pixels in the images.

## 3 Results and Discussion

**FIGURE 2:**
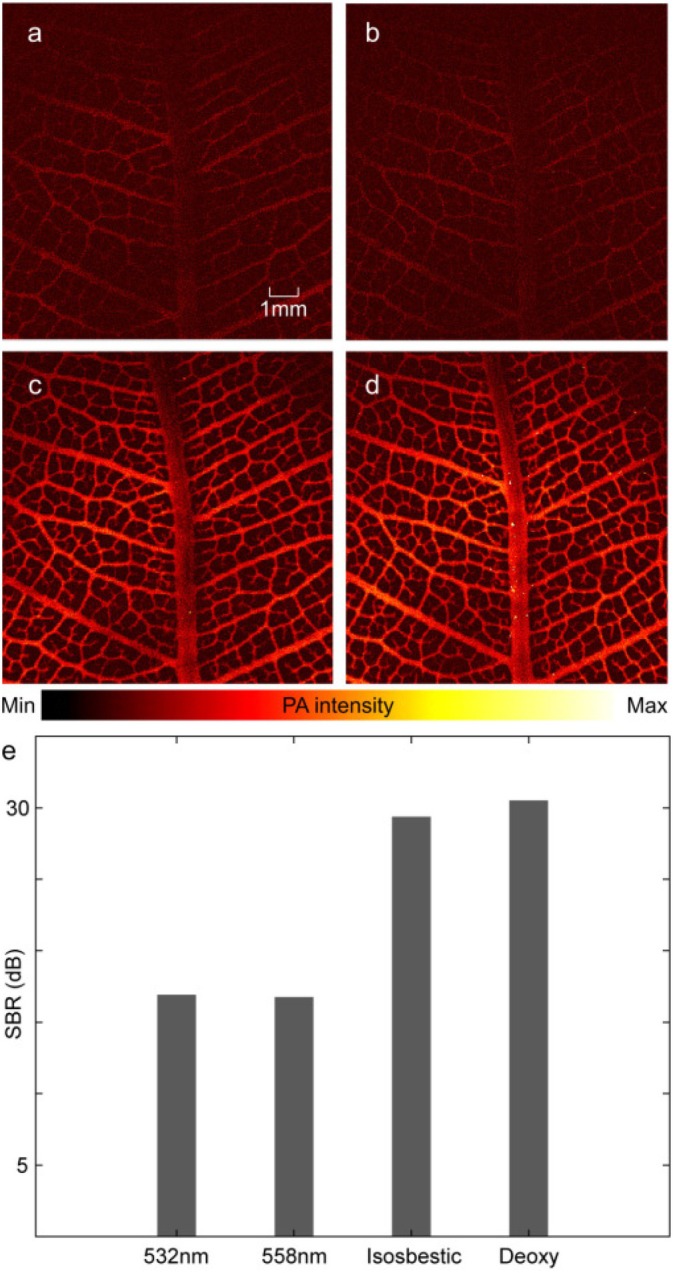
Leaf phantom imaging. Images acquired at (a) 532 nm (b) 558 nm, (c) isosbestic group (532 nm + 545 nm), (d) deoxyhemoglobin dominant group (Deoxy, (545 nm + 558 nm)), (e) bar chart showing comparison between the SBR of images shown in (a–d).

The system was initially characterized for image quality using a leaf phantom dyed with black ink (Black Skeleton Leaf, Etsy, New York, USA). Images were acquired using individual wavelengths—532 nm (Figure 2a) and 558 nm (Figure 2b)—as well as using grouped wavelengths corresponding to the isosbestic group (532 nm + 545 nm, shown in Figure 2c) and the deoxyhemoglobin‐dominant group (545 nm + 558 nm, shown in Figure 2d). Image quality was evaluated using SBR. A significant signal enhancement was observed when transitioning from individual wavelengths to grouped wavelengths (improving from 18 dB to approximately 32 dB). This improvement is attributed to the increased output energy when wavelengths are paired. For instance, the energy output increased from 120 ± 14 nJ at 532 nm to 285 ± 21 nJ for the isosbestic group, and from 124 ± 16 nJ at 558 nm to 291 ± 24 nJ for the. deoxyhemoglobin‐dominant group. The increase in energy proportionally amplified the photoacoustic signal.

**FIGURE 3:**
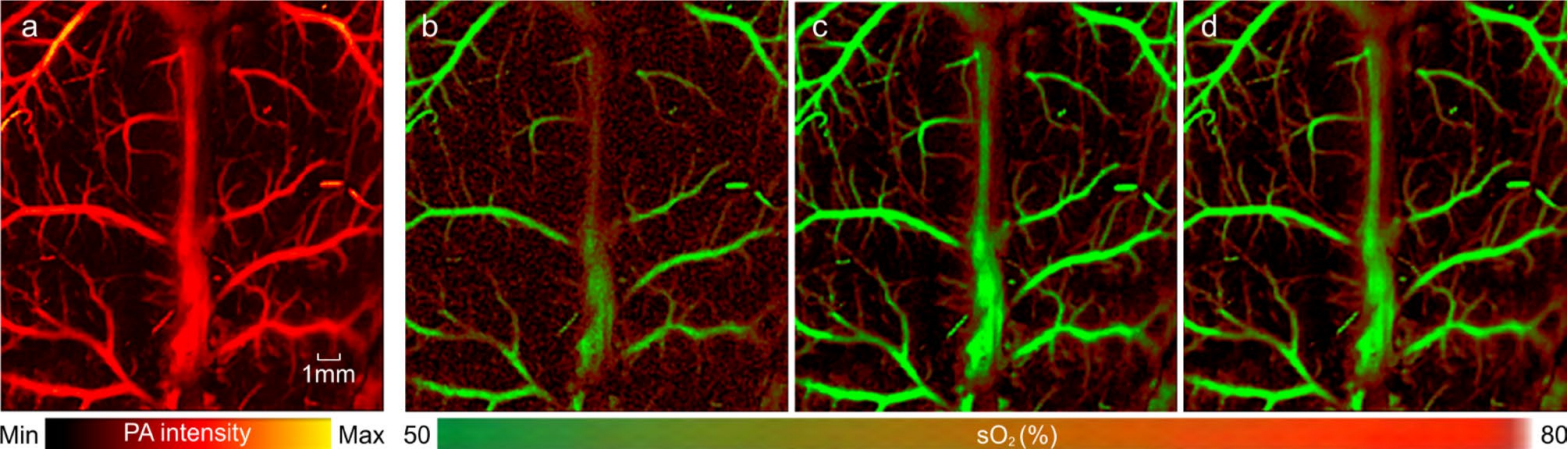
Validation of sO_2_ measurements. Images acquired in vivo from mouse brain using (a) maximum intensity projection (b) sO_2_ maps using the combination of the isosbestic (532 nm + 545 nm) group without averaging (c) with averaging and (d) deoxyhemoglobin‐dominant (545 nm + 558 nm) group with averaging. For fair comparison and compensation of difference in SBR, images in (c) and (d) are averaged 20 times and 5 times, respectively. sO_2_, oxygen saturation.

Next, we evaluated the performance of our developed system for mapping sO_2_. The formula to calculate sO_2_ is shown in Equation (1). In vivo images of the brain of a mouse were acquired, and sO_2_ maps were generated. Figure 3a presents the maximum amplitude projection (MAP) images of the mouse brain. Figure 3b shows the sO_2_ maps obtained using the 532 nm and 558 nm wavelengths without averaging, while Figure 3c shows the sO_2_ maps using the 532 nm and 558 nm wavelengths but with 20 times averaging. Figure 3d shows the sO_2_ maps generated using the grouped wavelengths method with 5 times averaging.

We empirically determined that we could obtain similar SBR from our wavelength‐grouped method and the traditional method if we averaged images acquired by our method 5 times, and images acquired by the traditional method 20 times.

We then calculated SSIM, NMSE and NCC to quantitatively evaluate the results (Table 1). The sO_2_ maps generated using 532 nm and 558 nm (the traditional method) served as the reference for calculating all quantitative metrics. SSIM achieved approximately 91.81% for all three biological targets. This high value shows that perceptually, our results are very similar to the traditional method. NCC, which is based on normalized pixel intensity, performed extremely well (around 96.83%). The NMSE of ~0.071 for the brain was very low, meaning the accuracy of the sO_2_ measurement per pixel is high. Overall, these results show the accuracy of our method for acquiring sO_2_ maps. While these values indicate good agreement with reference measurements, they are not perfect. This is expected due to several contributing factors. First, physiological variability such as spontaneous breathing can cause fluctuations in blood oxygenation over time, introducing dynamic changes during image acquisition. Second, respiratory motion and minor animal movements can introduce alignment errors between frames. Third, variations in laser pulse energy can lead to photoacoustic signal fluctuations, affecting the consistency of multispectral data. These limitations highlight areas for improvement in the current LS‐MS‐PAM setup. Several alternative approaches have been investigated to improve sensitivity in photoacoustic microscopy systems. For instance, deep learning–based reconstruction and denoising algorithms, when trained on sufficient datasets, can significantly enhance image contrast and signal‐to‐noise ratio [27, 28]. Meanwhile, double‐illumination PAM (DI‐PAM) adopts top and bottom‐side light delivery to improve penetration depth and light fluence distribution, achieving deeper imaging without exceeding ANSI limits [29]. Compressed sensing and related signal processing methods offer another route to reconstruct high‐quality images from sparse or subsampled data [27, 30, 31]. In contrast to these computationally or hardware‐demanding strategies, our LS‐MS‐PAM system offers a more direct and practical solution that enhances sensitivity while maintaining a large field of view. In future iterations, we plan to incorporate electro‐optical modulators for rapid and automated wavelength switching to minimize acquisition delays. We will also explore real‐time motion correction strategies and adopt focused or dual‐element transducers to enhance resolution and sensitivity. The fast optical scanning capabilities and large field of view of LS‐PAM, and now higher sensitivity of the method, make it highly suitable for functional brain imaging in small animals, a task that demands both high frame rates (> 1 Hz) and coverage of the entire cortical region. Another promising application is the imaging of tumor hypoxia, where subtle, localized fluctuations in oxygenation levels serve as critical biomarkers for both diagnosis and prognosis [6, 24, 32].

**TABLE 1:** Quantitative assessment of sO_2_ in the mouse brain.

SSIM (%)	NCC (%)	NMSE
91.81	96.83	0.071

Abbreviations: NCC, normalized cross‐correlation; NMSE, normalized‐mean‐square error; SSIM, structural similarity index matrix.

## 4 Conclusion

In this study, we developed and validated a novel wide‐field multispectral laser‐scanning photoacoustic microscopy system for measuring sO_2_ in vivo. By employing grouped wavelengths—specifically an isosbestic group (532 nm + 545 nm) and a deoxyhemoglobin‐dominant group (545 nm + 558 nm)—we significantly enhanced the SNR of the acquired signals. Phantom experiments demonstrated a remarkable SNR improvement from 18 dB (single wavelengths) to approximately 32 dB using grouped wavelengths, correlating with an energy increase from ~120 nJ to ~290 nJ per pulse. This enhancement enabled high‐quality sO_2_ mapping with reduced frame averaging. In vivo brain imaging in mice validated the accuracy of this method. Quantitative assessment yielded a SSIM of 91.81%, NCC of 96.83%, and NMSE of 0.071, all benchmarked against conventional two‐wavelength sO_2_ mapping. Notably, our grouped‐wavelength method required only 5× averaging to achieve comparable image quality to 20× averaging in the traditional approach, thereby improving temporal efficiency and reducing motion artifacts. Overall, this enhanced LS‐MS‐PAM system offers a highly sensitive, high‐throughput platform for functional imaging applications such as brain mapping and tumor hypoxia assessment. Future developments will focus on integrating rapid wavelength switching and motion correction to further boost its clinical and preclinical utility.

## Conflicts of Interest

The authors declare no conflicts of interest.

## Data Availability

The data that support the findings of this study are available from the corresponding author upon reasonable request.
